# 1768. Engaging Clinically and Socially Vulnerable Populations to Understand Their Perceptions of mRNA Vaccines

**DOI:** 10.1093/ofid/ofad500.1599

**Published:** 2023-11-27

**Authors:** Chelsea D’Silva

**Affiliations:** 19 to Zero, Oshawa, Ontario, Canada

## Abstract

**Background:**

One of the COVID-19 pandemic’s greatest silver linings has been the success of mRNA-based vaccines. While successful, the public has had many questions and even concerns about these vaccines as the pandemic is the first time they were clinically used. Both clinically and socially vulnerable populations have borne a hugely disproportionate burden of the pandemic. It is a public health imperative to ensure vaccine uptake in these groups are as high as possible. This project aimed to understand vulnerable populations perspectives on mRNA vaccines and immunizations to inform the development and dissemination of educational materials.

**Methods:**

We conducted semi-structured qualitative descriptive interviews with clinically vulnerable (e.g., 50+, those living in LTC and congregate settings, having immunocompromising conditions/treatments) and socially vulnerable (e.g., racialized, newcomer and indigenous) populations across Canada. We thematically analyzed the data to identify patterns of meaning across the interviews.

**Results:**

Fourteen people participated in interviews. Themes include: 1) clinically vulnerable populations view mRNA vaccines as safe and important for protecting themselves against COVID-19 and wish for the general public to get vaccinated to protect those who are most vulnerable; 2) socially vulnerable populations felt that there was a lack of accessible and concise information about who is high risk for COVID-19, timing of vaccination and how natural immunity to COVID-19 impacts the need for vaccination; and 3) vulnerable populations felt that the general public no longer views getting vaccinated as important since they do not view themselves as at-risk of hospitalization or severe adverse events.

**Conclusion:**

These findings suggest that vulnerable populations view the vaccine as safe but perceive that getting vaccinated is not a priority for the general public. Our team is developing educational resources for vulnerable populations and the public to promote vaccine uptake. With more mRNA vaccines being developed for other infectious diseases, these findings can help inform vaccine promotion efforts and increase confidence in mRNA vaccines.

**Disclosures:**

**All Authors**: No reported disclosures

